# Microscopic and Macroscopic Characterization of Hydrogels Based on Poly(vinyl-alcohol)–Glutaraldehyde Mixtures for Fricke Gel Dosimetry

**DOI:** 10.3390/gels10030172

**Published:** 2024-02-28

**Authors:** Silvia Locarno, Paolo Arosio, Francesca Curtoni, Marco Piazzoni, Emanuele Pignoli, Salvatore Gallo

**Affiliations:** 1 Dipartimento di Fisica “Aldo Pontremoli”, Università degli Studi di Milano, Via G. Celoria 16, 20133 Milano, Italy; paolo.arosio@unimi.it (P.A.); franscesca.curtoni@studenti.unimi.it (F.C.); marco.piazzoni@unimi.it (M.P.); salvatore.gallo@unimi.it (S.G.); 2 Istituto Nazionale di Fisica Nucleare (INFN), Sezione di Milano, Via G. Celoria 16, 20133 Milano, Italy; 3 Consorzio Interuniversitario Nazionale per la Scienza e Tecnologia dei Materiali (INSTM), 20133 Milano, Italy; 4 Fondazione IRCCS “Istituto Nazionale dei Tumori”, Via G. Venezian 1, 20133 Milano, Italy; emanuele.pignoli@istitutotumori.mi.it

**Keywords:** poly(vinyl-alcohol) hydrogel, crosslinked network, gel characterization, NMR-relaxation, gel-dosimetry

## Abstract

In recent decades, hydrogels have emerged as innovative soft materials with widespread applications in the medical and biomedical fields, including drug delivery, tissue engineering, and gel dosimetry. In this work, a comprehensive study of the macroscopic and microscopic properties of hydrogel matrices based on Poly(vinyl-alcohol) (PVA) chemically crosslinked with Glutaraldehyde (GTA) was reported. Five different kinds of PVAs differing in molecular weight and degree of hydrolysis were considered. The local microscopic organization of the hydrogels was studied through the use of the ^1^H nuclear magnetic resonance relaxometry technique. Various macroscopic properties (gel fraction, water loss, contact angle, swelling degree, viscosity, and Young’s Modulus) were investigated with the aim of finding a correlation between them and the features of the hydrogel matrix. Additionally, an optical characterization was performed on all the hydrogels loaded with Fricke solution to assess their dosimetric behavior. The results obtained indicate that the degree of PVA hydrolysis is a crucial parameter influencing the structure of the hydrogel matrix. This factor should be considered for ensuring stability over time, a vital property in the context of potential biomedical applications where hydrogels act as radiological tissue-equivalent materials.

## 1. Introduction

Hydrogels are hydrophilic three-dimensional (3D) polymer networks capable of retaining a significant amount of water without dissolution, thanks to the chemical and/or physical crosslinking of individual polymer chains. In particular, the ability of hydrogels to absorb water and, at the same time, resist dissolution arises from the hydrophilic functional groups of the polymeric backbone and from the crosslinking density between the network chains. Thanks to the extraordinary swelling/deswelling capacity caused by the movement of the water inside the structure, non-biodegradable hydrogels are able to maintain good mechanical stability over time. In other words, a hydrogel matrix should ensure physical and mechanical integrity to prove effective in its use as biomaterials during the lifetime of an application [1,2]. Because of the advances in hydrogel technologies and their versatile fabrication methods and tunable physical properties, hydrogel materials have been applied in a wide range of biomedical and engineering applications, ranging from cell culturing [3] to tissue engineering [4,5,6], sensors and actuators [7,8], drug delivery [9,10], and so on. In particular, this work focuses on the characterization of hydrogels used for dosimetric applications and, specifically, Fricke gel (FG) dosimetry. The Fricke gel dosimeter is one of the most common chemical gel dosimeters used in radiation therapy. It mainly consists of ferrous ions (Fe^2+^), which constitute the radiation-sensitive species dispersed in a 3D tissue-equivalent matrix. Upon radiation exposure, Fe^2+^ ions are converted into ferric (Fe^3+^) ions as a function of the absorbed radiation dose. The Fe^3+^ ions formed can be evaluated, for example, using optical techniques, nuclear magnetic resonance (NMR), or magnetic resonance imaging (MRI) [11,12,13,14].

For many years, the two conventional hydrogels used in FG dosimetry were based on gelatin and agarose [11]. Later, matrixes based on synthetic Poly(vinyl-alcohol) (PVA) replaced them because, unlike organically based materials, this polymer is obtainable with tight manufacturing tolerances and with low impurity levels; therefore, the starting product is more homogenous than those products of previous methods [15,16,17].

PVA is a common water-soluble synthetic polymer with excellent basic properties and a simple chemical structure and, beyond FG dosimetry, has great potential applications in many fields. In fact, thanks to its biocompatibility, low tendency towards protein adhesion, low toxicity, and cheapness, PVA is used for several applications in the food industry and forestry and as a super absorbent, but most of all, it is used in biomedicine for drug delivery purposes, tissue engineering scaffolds, biosensors, wound dressings, and soft robotics [18].

PVA hydrogels are usually realized via physical or chemical crosslinking. The principle of physical crosslinking is based on the intermolecular interactions that are, for example, accomplished with the aid of freeze–thaw cycles. The obtained cryogel, however, is inhomogeneous and not completely transparent, ranging from translucent to completely opaque. The inhomogeneities become macroscopic when cryogel samples of consistent volume are produced, and, from the dosimetric point of view, such cryogels showed lower radiation dose sensitivity compared to conventional FGs [15,16,17].

**Scheme 1:**
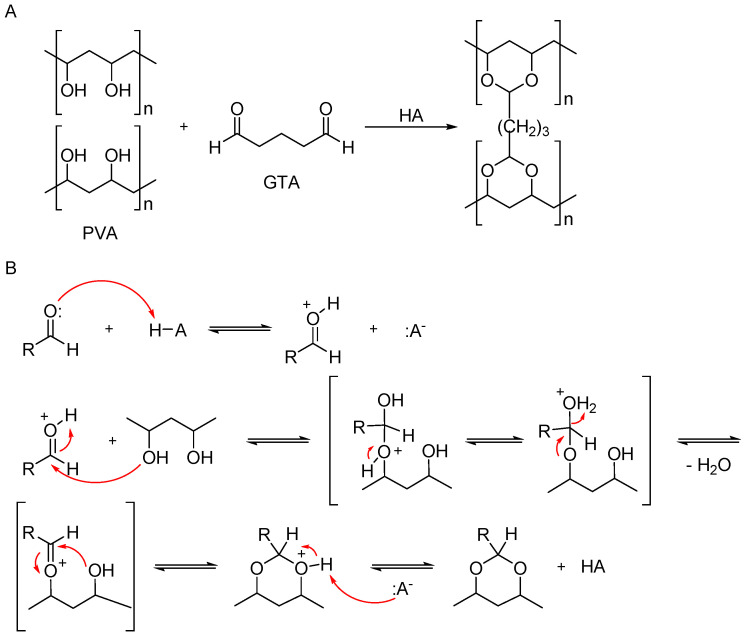
(**A**) Crosslinking reaction between PVA and GTA. (**B**) Mechanism of the acetylation reaction between a hydroxyl-containing polymer and an aldehyde.

Alternatively, PVA gel formation could be achieved via chemical pathways using Glutaraldehyde (GTA) as a crosslinking agent. [19] The mechanism of the reaction between PVA and GTA is described in Scheme 1. Under acidic conditions, the acetylation reaction occurs at room temperature and leads to the formation of acetal rings along the polymer chain. The formed hydrogel appears transparent, and it is possible to modulate its characteristics in a reproducible way by varying the concentration of PVA, its molecular weight, and GTA/PVA ratio.

PVA-GTA-FG dosimeters have demonstrated comparable radiation dose sensitivity to conventional FGs but with lower ion diffusion [20,21,22,23]. Moreover, chemical crosslinking increased the rigidity, transparency, and thermos-stability of the matrix, and, consequently, produced solid dosimeters for 2D and 3D optical detection methods. For these reasons, over the years, PVA-GTA-based hydrogels have become the matrix of choice for the development of FG dosimeters. However, the absence of a standard formulation for the preparation of these systems induced research groups to use PVA with different chemical features (such as molecular weight, degree of hydrolysis, impurity content, etc.) on the basis of their availability [24,25,26,27,28,29,30,31].

Therefore, the aim of this work is to compare PVA-GTA hydrogels produced with different types of PVA in order to find out any differences in behavior that could affect their applications. The collected data are interesting not only in the field of gel dosimetry but also in relation to several general biomedical uses.

In particular, driven by the results shown in the work of Lazzeri and coworkers [22], additional studies concerning the microscopic, macroscopic, and dosimetric properties of PVA-GTA-FG dosimeters have been carried out, investigating the influence of PVA molecular weight (MW) and hydrolysis degree (HD) on the physical–chemical properties, mechanical characteristics, and dosimetric response of gels.

The macroscopic studies were combined with ^1^H-NMR relaxometric measurements that report the interactions of nuclear spins of water and polymers hydrogens between them and with the local surrounding environment (like chemical functional groups, electron density, etc.). Finally, optical absorbance spectral analyses of PVA-GTA gels loaded with Fricke solution were conducted to characterize their response with respect to dose within the typical intervals relevant to external X-ray radiation therapy.

## 2. Results and Discussion

### 2.1. Sample Preparation and Viscosity Determination

**Figure 1:**
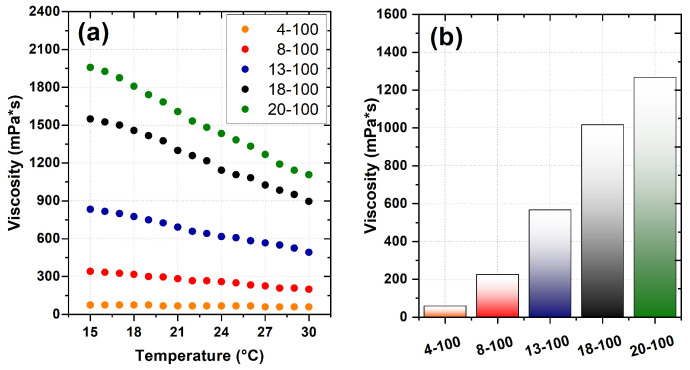
(**a**) Viscosity profile of PVA stock solutions at increasing temperature and (**b**) viscosity values of PVA stock solutions at 27 °C.

PVA stock solutions were prepared by dissolving PVA powder in water at 12.4% *w*/*w* concentration. Then, PVA solutions were mixed according to the proportions reported in Table 6 (Section 4). Before the gelation of the samples, the viscoelastic properties of the PVA mixture solutions were analyzed. First of all, the viscosity of the PVA solutions was measured by setting the fixed shear speed and increasing the temperature from 15 to 30 °C. For all samples, the viscosity decreased with increasing temperature, as shown in Figure 1a and Figure S1. In fact, at higher temperatures, the molecular vibrations are greater and, consequently, the intermolecular interactions are less stable and, therefore, weaker than at lower temperatures. Then, the viscosity of the stock solutions at 27 °C was compared (Figure 1b). As expected, the viscosity of the solutions increased with the increasing molecular weight (MW) of the PVA (*4-100* > *8-100* > *13-100* > *18-100*) because larger molecules encounter more resistance to movement compared to smaller molecules. The viscosity measurement of the polymer solution is, in fact, a common strategy used to determine the size of the polymer, leading to the chain length and MW.

The presence of intermolecular interactions is also crucial in determining how tightly two molecules hold onto each other and, consequently, their resistance to flow. This is evident when comparing *18-100* solution with *20-100* solution, which have similar MWs but a different hydrolysis degree (HD, 88 and 98%, respectively). Figure 1b shows that the *18-100* solution has a lower viscosity than *20-100*. A HD of 88% means that 12% of the hydroxyl groups (-OH) of the PVA chain are in acetylated form; the remaining 88% are in free form. In *20-100* solution, only 2% of the -OH group is in an acetylated form. -OH groups are able to make strong hydrogen bonds with water molecules, so the greater the number of free -OH groups in the polymer chain, the stronger the intermolecular interactions and, therefore, the resistance to flow increases.

**Figure 2:**
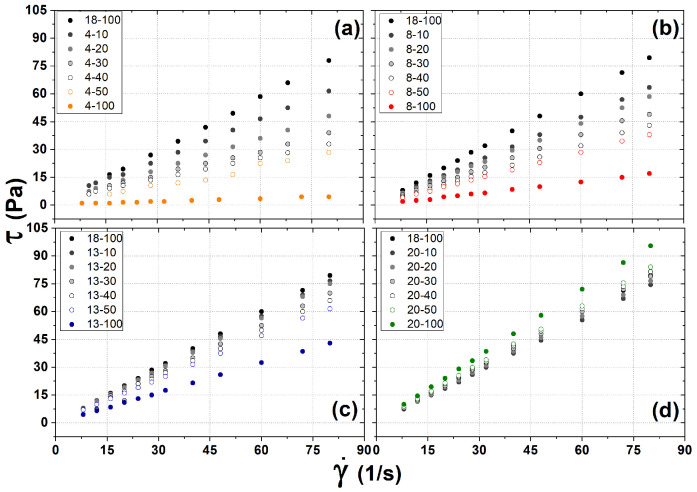
Viscosity profile of PVA mixture solutions at different shear rates at 27 °C for the different PVA mixtures: (**a**) *4-*, (**b**) *8-*, (**c**) *13-*, (**d**) *20-* mixtures, respectively.

Figure 2 shows the shear stress (τ) as a function of a shear rate ($\dot{\gamma }$) for all the PVA mixture solutions, fixing the temperature at 27 °C. This plot displays a linear increase in stress with increasing shear rate, suggesting a Newtonian property for all the PVA solutions regardless of MW and HD. According to Newton’s laws, the viscosity η of PVA solutions, as given by the slope of the plotted line, remains constant independent of the rate of shear and, as already observed in Figure 1b, it only depends on the temperature. The viscosity analyses also showed that the viscosity of the PVA solution can be finely modulated by mixing PVA with different MWs and HDs (Figure 2 and Figure S1).

The PVA mixture solutions were then diluted with acidic water until reaching a PVA concentration of 9.1% *w*/*w*, and finally, GTA was added to induce gelation. Then, the mechanical and physical–chemical characterization of the different PVA hydrogel samples was performed with the aim of elucidating the role of MW and HD of PVA on the determination of hydrogel properties.

### 2.2. Mechanical Characterization of Hydrogels

The mechanical behavior of PVA hydrogels was assessed by compressing the samples at a fixed rate (10 mm/min) at room temperature (25.0 ± 0.5 °C). As expected, the magnitude of Young’s modulus (*E*) increased with the MW of the PVA. E values are reported in Table 1 and are within the same order of magnitude as those found in other similar studies focusing on PVA-based hydrogels [32,33,34]. Indeed, as polymeric chains become longer, they also become more entangled, thus conferring strength to the material [35]. This is indicative of stronger inter- and intra-chain interactions.

**Figure 3:**
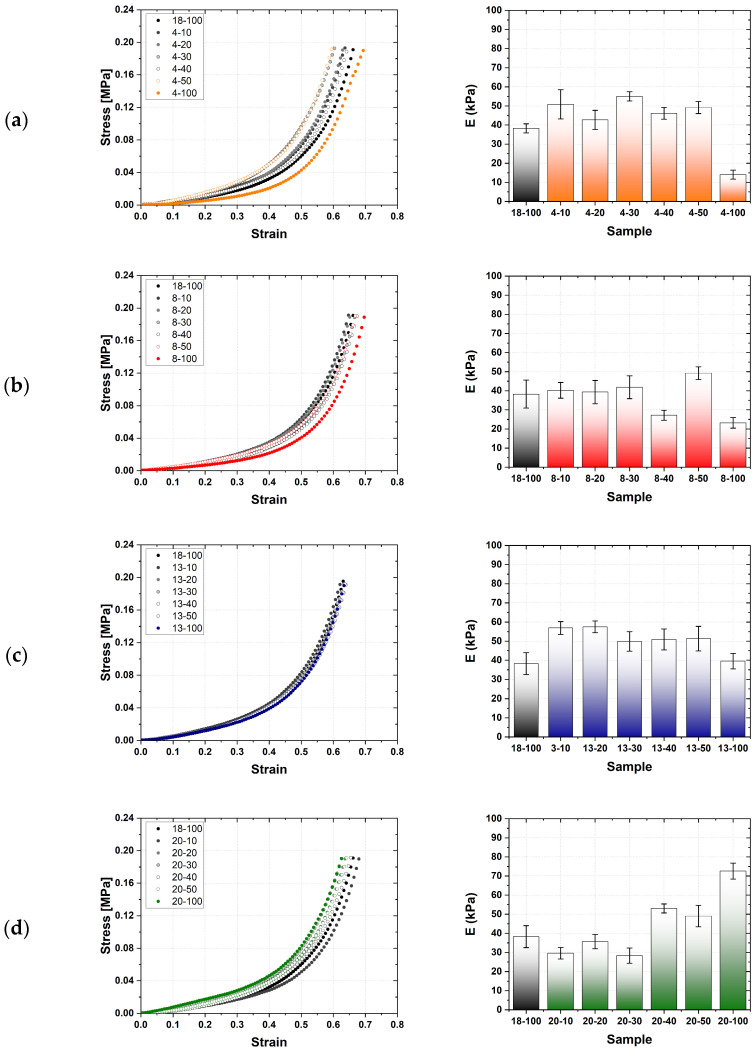
Uniaxial compressive tests on PVA hydrogels. Stress–strain curves (**left**) and Young’s Moduli (**right**) of (**a**) *4-*, (**b**) *8-*, (**c**) *13-*, and (**d**) *20-* mixtures.

Stress–strain curves of all PVA hydrogels (Figure 3) displayed the typical shape of polymeric elastic materials, with a linear region up to ~30% of the undeformed samples’ height at relatively low stress (<0.05 MPa). Despite the onset of plasticization, which is depicted by the non-linear portion of the curve (strain > 30%), none of the samples fractured at the macroscale within the analyzed loading range (40 N).

However, differences in the evolution of Young’s modulus as a function of the PVA hydrogel mixing ratio can be pointed out. Series *4-* (Figure 3a) and *8-* (Figure 3b) PVA samples did not show a continuous decrease in mechanical strength by increasing the amount of 4:88 and 8:88 PVA in the mixture. Rather, it seems that the PVA with the highest MW (18:88) has a predominant effect in determining the overall Young’s modulus of the hydrogel with respect to the PVAs with lower MWs (4:88 and 8:88). This can also be evidently seen from the stress–strain plots, where the curves of the *-10*, *-20*, *-30*, *-40*, and *-50* samples gathered around that one of sample *18-100*.

On the other hand, the *13*- series mixtures (Figure 3c) did not show significant differences in terms of Young’s moduli at any mixing ratio, probably because they were made of PVA with more comparable MWs.

A completely different trend was observed for hydrogels composed of 18-88/20-98 PVA mixtures (Figure 3d). In this case, a marked correlation between the mechanical behavior of the specimens and the PVA mixing ratio can be seen, as Young’s modulus was found to increase more smoothly by increasing the 20:98 PVA content.

This behavior can be explained by the fact that 20:98 PVA possesses a higher HD than the other PVA taken into consideration in this study. Therefore, after crosslinking with GTA, samples with a higher content of 20:98 PVA will also have a higher crosslinking density (i.e., the number of effective crosslinks per unit volume [36]), which will ultimately result in a tougher material [37]. In conclusion, it is safe to attest that variations in the MW and HD of the precursor PVA polymer solution can be a useful predictive tool to selectively tune Young’s modulus of the chemically crosslinked hydrogels.

### 2.3. Physical–Chemical Characterization of the Hydrogels

The chemical nature of the gel samples was investigated through FTIR analysis. Figure S2a shows the FTIR spectra of the PVA powders used for the preparation of the hydrogel samples. Five characteristic regions were identified [38,39]. The strong large absorption peak at around 3300 cm^−1^ is related to the O-H stretching from the intramolecular and intermolecular hydrogen bonds and, consequently, it is more intense in PVA 20:98, which has a higher HD (thus more free –OH groups). The vibration band at around 2900 cm^−1^ is linked to the C-H stretching from alkyl groups, while the sharp peak around 1700 cm^−1^ refers to the C=O and C-O stretching from acetate groups; in fact, in PVA 20:98, this peak is less intense. The bands at around 1250 cm^−1^ and 1083 cm^−1^ are attributed to the C=O vibration from acetate groups and C-O stretching in the C-O-H groups, respectively [40]. For this reason, in PVA 20:98, the first peak is less intense, while the second one is more intense.

Subsequently, in order to verify that the hydrogel samples were corrected crosslinked, FTIR analyses of the hydrogel samples were performed and compared with the powder spectra (Figure S2b). The FTIR spectra of the PVA-GTA-based hydrogels show a duplet absorption at 2850 cm^−1^ and an important peak at 2730 cm^−1^, which are attributed to the C-H stretching band related to the formation of acetal rings. The reduction in the intensity of O-H peaks is particularly evident for sample *20-100*, suggesting a greater number of acetal bridges [41]. Moreover, with the increasing HD of PVA, the broad peak at 3300 cm^−1^, assigned to the O-H stretching vibration due to the hydrogel bonds, shifted to 3285 cm^−1^ and 3277 cm^−1^ for samples *20-50* and *20-100*, respectively. The redshift of the peak indicates that the number of hydrogen bonds between polymer chains increases in hydrogels with higher HD [42]. Finally, in the hydrogel spectra, a shoulder appears around 1690 cm^−1^, which is attributable to the C-O stretching of the acetal portion.

**Figure 4:**
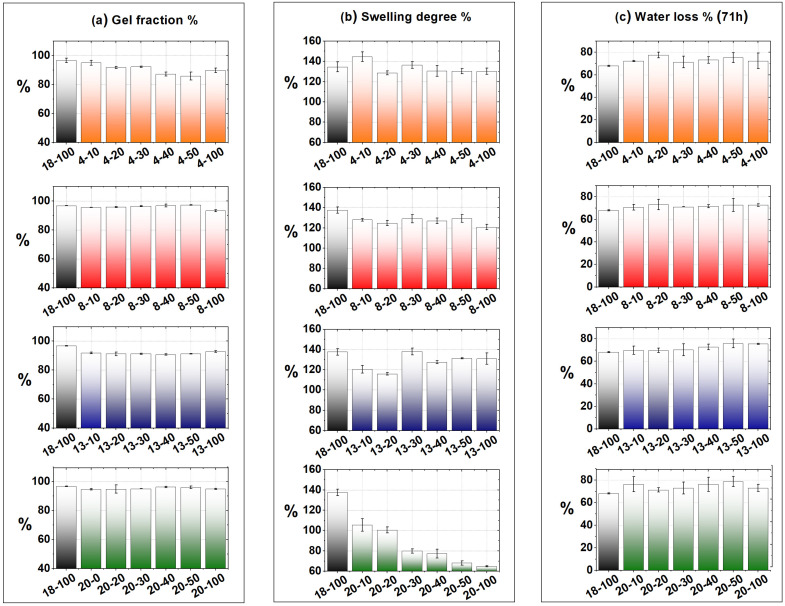
(**a**) GF%, (**b**) SW%, and (**c**) WL% after 71 h of preparing the PVA hydrogel samples.

From the macroscopic point of view, the ability of PVA hydrogels to absorb water arises from -OH groups of the polymer backbone, while their resistance to dissolution arises from crosslinks between polymer chains [43]. These two parameters can be studied through the estimation of SW% and GF% coefficients (Figure 4a,b).

As shown in Figure 4a, the GF% coefficient is independent of both the MW of the PVA and its HD. For all PVA-GTA gel samples, the GF% reached a value of 95% on average, with variations lower than 5% between the analyzed samples, indicating that all the PVA molecules became connected in the gel network.

On the other hand, swelling capacity is one of the basic characteristics of hydrogels, and it is determined by the amount of space inside the hydrogel network that is available to accommodate water. In the case of PVA-based hydrogels, which are non-ionic hydrogels, the SW% is exclusively determined by the result of polymer–water interaction forces. These interactions are affected by many factors, such as the nature of the solvent, polymer structure, network parameters, and drying techniques [44]. However, the most important factor is the crosslinking density of the hydrogel network, which is determined by the distance between two crosslinks in the same polymer chain. The shorter their distance, the higher the crosslinking density [45]. SW% data are shown in Figure 4b. Only PVA hydrogels containing PVA 20:98 displayed a significant variation in SW% compared to other PVA-based hydrogels analyzed. In particular, when increasing the percentage of PVA 20:98, the swelling capacity of the gel decreased.

This can be explained, once again, by the fact that PVA 20:98 possesses a high HD; in fact, 98% of the –OH groups are available for the crosslinking reaction with GTA and, consequently, the crosslinking density is greater than that of the hydrogel formed with PVA, with a HD of 88%. An increase in crosslinking density results in decreased swelling since the water diffusion is hindered by the limited deformation process of the polymeric network. Otherwise, the crosslinking density is independent of the MW of PVA since the SW% of hydrogels containing PVA 4:88, 8:88, 13:88, and 18:88 is almost the same with a value of 130% and a statistical variation of 6% between the samples.

Unlike the SW% coefficient, which measures the water bound in the deep layers of the hydrogel, the WL% coefficient refers to water evaporation at the surface level. The water accommodated by the hydrogel can be classified into three types. The free water in the outermost layer is easily removed under mild conditions (e.g., mechanical compression), while interstitial water is physically trapped in the polymeric network and has much weaker interactions than bound water [44]. The evaporation of the bound water depends on polymer–water interactions and, consequently, how the water can reach the surface moving inside the gel via capillary action or diffusion. Figure S3 shows the evaporation profiles of PVA hydrogel samples as a function of time, while Figure 4c reports the percentage of water lost by the hydrogel after 71 h at room temperature.

The data suggested that polymer–water interactions were not affected by either the MW or the HD of the PVA since the WL% coefficients did not show any significant variations (less than 3%) between the samples.

In order to confirm these data, contact angle measurements were carried out using the water drop method. The images and the contact angles in degrees are reported in Figure S4 and in Table 2. Additionally, in this case, in general, no significant variations were found between the samples, confirming the previous results of the WL% coefficients. Only *4-100* and *4-50* samples containing PVA with the lowest MW (4:88) showed a slight decrease in the contact angle value. This suggests that bound water inside these hydrogels is able to reach the surface more easily, probably due to the greater softness of such gels (see Table 1). For other samples, the calculated mean contact angle found is between 50 and 60°, which is in line with the results reported in the literature for PVA hydrogels [46]. The variation between the samples could be attributed to an inhomogeneous water evaporation degree in the samples rather than a different polymer–water interaction behavior.

### 2.4. ^1^H NMR Relaxometry Measurements

The above macroscopic characterization of the hydrogel mixtures induced us to investigate their local behavior using broadband ^1^H NMR relaxometry measurements. The longitudinal nuclear relaxation time T_1_ and the transverse nuclear relaxation time T_2_, two parameters related to the relaxation process of hydrogen nuclear magnetization, were measured. These parameters depend on the interaction between the hydrogen nuclei and the local surrounding environment (i.e., all the other factors able to interact with the spins of the hydrogen nuclei), which, in our case, can be electron density, chemical functional groups, and other hydrogen nuclei. It must be noted that in the samples used for these measurements, most hydrogen nuclei are water protons; therefore, most of the obtained NMR signals reflect the information coming from the water molecules inside the samples, and the residual signal comes from the hydrogen nuclei of the polymers.

With the above in mind, *4-100*, *8-100*, *13-100*, *18-100*, *20-100*, *4-50*, *8-50*, *13-50*, and *20-50* samples were investigated by analyzing the relaxation curves of the longitudinal and transverse nuclear magnetization obtained at ν = 31.6 MHz.

In the case of longitudinal relaxation T_1_ (also called spin-lattice nuclear relaxation time), all of the PVA samples can be considered to have the same reported interaction time in terms of hydrogen nuclear spins with almost only water molecules in the surrounding environment (chemical functional groups and the electron density of the hydrogels). The estimated T_1_ value for each sample is reported in Table 3 (the experimental longitudinal relaxation curves are included in the supporting information in Figure S5). The relative mean times and their standard error extrapolated from the above T_1_ are 1.506 and 0.023, respectively.

**Figure 5:**
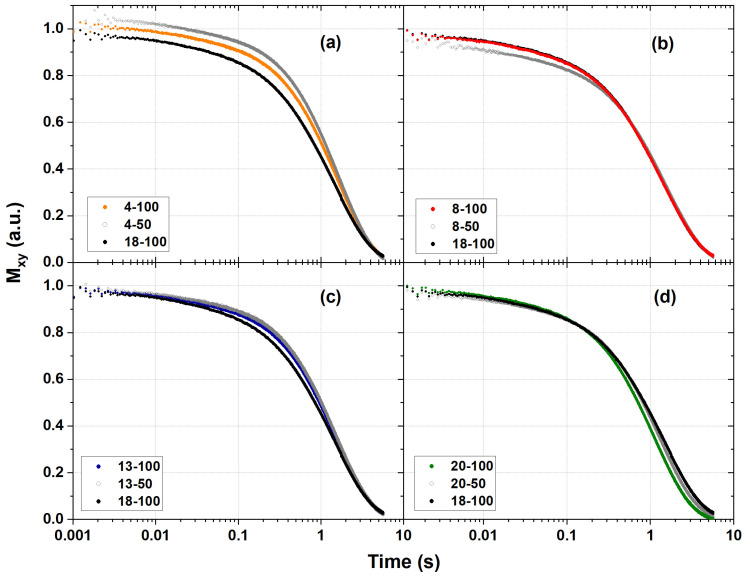
Transverse nuclear relaxation decay curves of the samples: (**a**) *4-100*, *4-50* and *18-100*; (**b**) *8-100*, *8-50* and *18-100*; (**c**) *13-100*, *13-50* and *18-100*; (**d**) *20-100*, *20-50* and *18-100*.

The transverse decays of all the samples are reported in Figure 5. When plotting the exponential decay in a semilog scale, as shown in Figure 5, with the first part of the curve zoomed, the fastest part of the decay is clearly shown. Indeed, the curves indicate the presence of more than one component that constitutes T_2_ exponential decay (also called spin–spin nuclear relaxation times).

In detail, some samples are characterized by two components of decay, while in others, three components can be recognized. We distinguished these components as follows: “T_2_ fast component” (T_2 fast_), “T_2_ medium component” (T_2 medium_), not always present, and the “T_2_ slow component” (T_2 slow_)m and their presence can be ascribed to the different environments that influence the nuclear relaxation of the hydrogen spins.

To fit the exponential decays, we used a fitting model with three components, as expressed by Equation (1):

(1) $$y=y_{0}+{w_{1}\cdot e}^{-\left(\frac{t}{T_{2\;fast}}\right)}+{w_{2}\cdot e}^{-\left(\frac{t}{T_{2\;medium}}\right)}+{w_{3}\cdot e}^{-\left(\frac{t}{T_{2\;slow}}\right)}$$

where *y*_0_ is almost zero, *w_i_* represents the weights of each component of the signal, and *T_i_* indicates the constant times of each component of the decays. The results obtained from this fitting procedure are reported in Table 4.

It should be noted that a major part of the signal (≥75–80% in all the curves) is due to the T_2 slow_ component, which is surely correlated to the hydrogen nuclei of water molecules. Indeed, taking into account the error in the estimation of time constants, the T_2 slow_ values are compatible with the T_1_ values presented in Table 4, except for *20-100* and *20-50* samples, because ^1^HNMR relaxometry is able to also detect also contributions caused by the different confinements of the water molecules (for instance [47,48]). Concerning the T_2 fast_ component, when considering their values (typical of relaxation times that pertain to spin atomic species interacting strictly with other neighbor nuclear spins) and the w_1_ values comparable to PVA content in the hydrogels, it is possible to assert that part of the decay curves can be assigned to the contribution of the PVA hydrogens. Conversely, the T_2 medium_ component, not always valuable in terms of our experimental data, could be tentatively attributed to the interaction between the hydrogen of water molecules and the nuclear spins of Poly(vinyl-alcohol).

**Figure 6:**
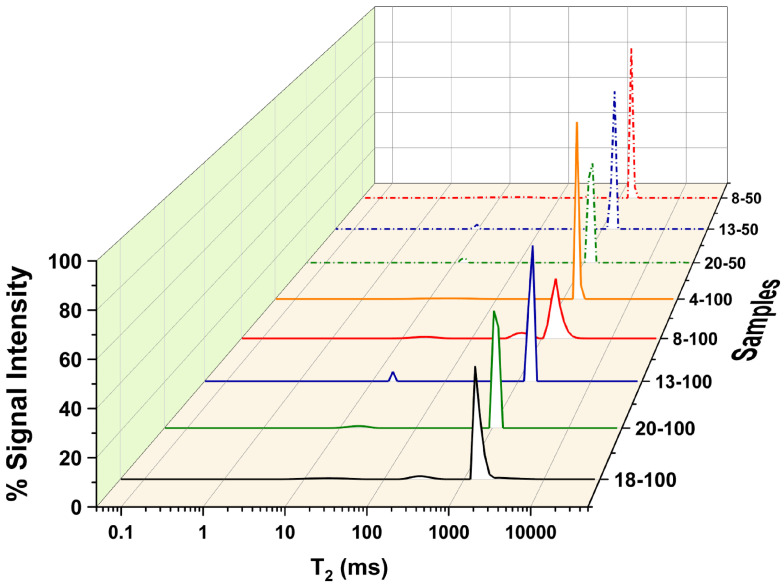
Inverted nuclear relaxation time distributions for all the samples obtained using UPEN.

In order to confirm the results of the fitting model, we processed the experimental spin–spin relaxation signals by means of the inverse Laplace transform using the UPEN algorithm, and the multi-exponential curves were inverted, and the obtained distributions of relaxation times were correlated to a different class of hydrogens present in the hydrogels (see Table 5 and Figure 6).

Both the procedures indicate that the T_2_ of all the samples can be described by a slow relaxation time with *1.1 s < t < 1.7 s* and a lower contribution to the relaxation due to one or two others relaxation times (a much faster one below 40 ms and, where present, a medium one of hundreds of milliseconds), see Table 4 and Table 5.

As already stated, the slow relaxation time values are really similar for all the samples except for the *20-100* and *20-50* samples that contain PVA 20:98—the samples with higher HD. In particular, the value of the T_2 slow_ *20-100* sample indicates a stronger interaction between the hydrogen spins of the water molecules inside the gel matrix for this sample in comparison to the other PVA hydrogel samples, where the interactions between spins (T_2_) and the spins and lattice (T_1_) are described by the same constant time values. Therefore, the relaxometry results are in agreement with the properties of the higher HD, viscosity, and crosslinking density of the *20-100* sample. The T_2 slow_ *20-50* sample with a T_2 slow_ value of about 1.3 s denotes a hydrogen spins behavior in the middle of the previous situations, as can be expected by mixing sample PVA 20:98 and PVA 18:88 with the characteristics just discussed. On the other hand, the inverse Laplace transform procedure also confirms the presence of the other two classes of hydrogen nuclei.

One class has quite fast relaxation times ascribable to PVA hydrogens with values comparable to those obtained through the fitting model, and a second class is difficult to detect in all the samples.

### 2.5. Dosimetric Analysis

**Figure 7:**
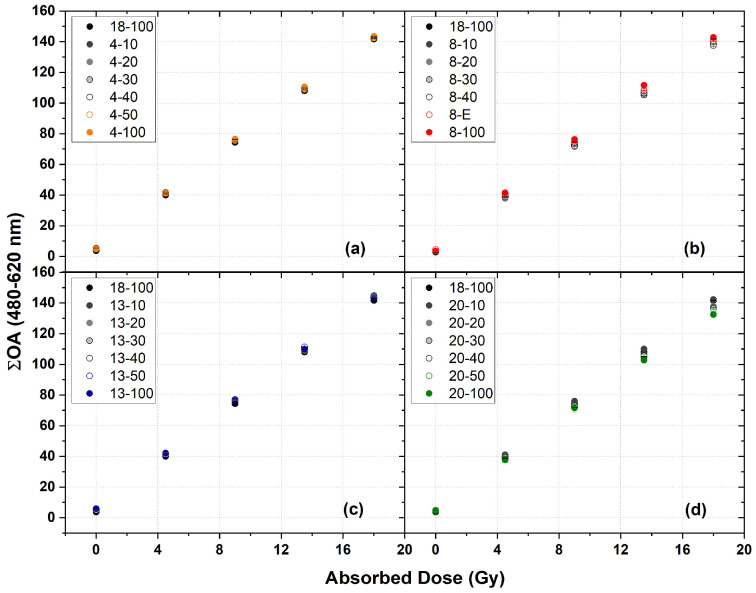
Cumulative OA versus dose for the different PVA (**a**) *4-*, (**b**) *8-*, (**c**) *13-*, and (**d**) *20-* mixtures. Error bars correspond to one standard deviation.

Figure 7 illustrates the trend of the cumulative optical absorbance versus absorbed dose for PVA-GTA-FG, arranged according to the PVA mixtures detailed in Table 6. The optical response consistently increases within the investigated dose range (0–18 Gy), aligning with the findings reported in the literature for an FG made with other PVA types [18,28]. Subsequently, the experimental data underwent linear regression analysis, where the slope of the fitted line served as an indicator of optical dosimetric sensitivity (see Table S1). Remarkably, a consistent linear trend was observed for all preparations, with an R^2^ value exceeding 0.999.

The sensitivity of PVA-GTA-FG, produced with PVA mixtures of 4:88, 8:88, 13:88, and 18:88, exhibited no discernible differences (Figure 7a–c). The maximum variability among preparations with 18:88 was less than 1.5% for any other PVA considered. Consequently, we assert that the MW of the polymer does not significantly influence the dosimetric response within the investigated range. These variations lack a definitive trend and fall within the scope of experimental variability.

Conversely, it was observed that for comparable MWs (PVA 18:88 and 20:98) but with different HDs, the dosimetric response is affected. Specifically, the dosimetric sensitivity decreases as the percentage of 20:98 increases in the mixture with 18:88, declining from 7.65 to 7.33. This variation exhibits a noticeable trend, with the FG obtained from PVA 20:98 demonstrating an optical sensitivity that is 5% lower than that of 18:88. Consequently, HD emerges as a key factor influencing the dosimetric response. This result advocates for the use of PVA with a partial HD in dosimetric applications.

As established in prior research, an auto-oxidation process takes place over time [26]. This phenomenon occurs continuously both before and after irradiation. Consequently, investigating the effects of thermal stress on unirradiated samples has become imperative. To address this, several PVA-GTA-FG samples underwent thermal stress, as detailed in Section 4.11.3. The analysis of these samples took place four hours after their preparation. The Σ(OA) values were then calculated from the oxidation spectra obtained at various intervals of thermal stress and at temperatures of 27.0 ± 0.5 °C.

**Figure 8:**
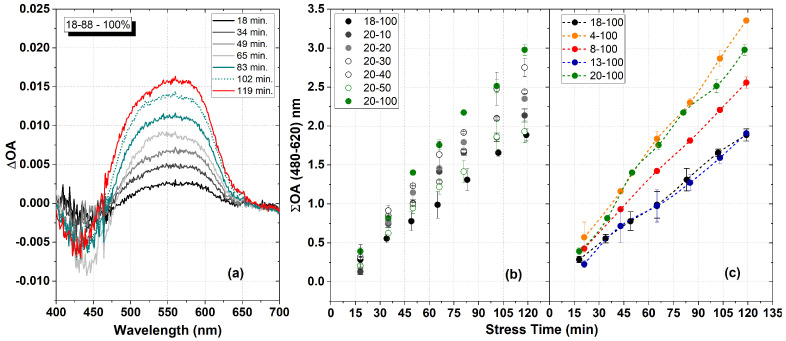
(**a**) Optical absorbance spectra were obtained for an unirradiated sample subjected to heat stress at 27 °C. The experimental Σ(OA) of unirradiated FGs versus stress-time for the different PVA mixtures (20-98 and 18-88) (**b**) and for different pure PVA (**c**). Each point is the mean of measurements on at least three samples. Error bars correspond to the standard deviation. The lines represent the weighted linear fits of the data.

An example of the Δ(OA) spectra for an unirradiated dosimeter acquired at different time intervals is shown in Figure 8a. The spectra refer to the mixture of pure PVA 18:88.

The appearance of the broad optical absorption band for wavelengths greater than 500 nm is evident. This optical absorption band is attributable to the formation of complexes of Fe^3+^ ions with XO. In this case, Fe^3+^ ions are not radio-induced but produced by thermal stress (auto-oxidation).

For each OA spectrum and for all the different preparations (Table 6), the Σ(OA) was calculated. For example, the results relating to the preparations of *18-100*, *20-10*, *20-20*, *20-30*, *20-40*, *20-50*, and *20-100* are reported in Figure 8b.

All of the experimental data were fitted with a linear function of a free intercept. Figure 8c shows the obtained results for the pure PVA-GTA-FG. It can be observed that for all of the considered preparations, as the stress time increases, the integral optical absorbance value increases.

It can be observed that all curves have intercepts comparable to zero, which is within the experimental error. The results show that MW affects the dosimetric response over time. In particular, the auto-oxidation rate is higher for PVAs with lower MWs. The results for mixtures of PVA 13:88 and 18:88 are comparable with each other within experimental variability. HD is also a parameter to consider. PVAs with comparable MWs and different HDs show different behaviors. Higher HD favors auto-oxidation. A systematic trend has been found in the mixtures. This study identified which chemical and physical parameters influence the dosimetry response of PVA-GTA-FG.

## 3. Conclusions

Hydrogels have been synthesized using PVA with various chemical features using the chemical crosslinker method. In particular, the influence of the MW and HD of the types of PVA used in the dosimetric field on the macroscopic, microscopic, and dosimetric features of hydrogels were investigated.

The overall results have shown that, based on the PVAs considered, at macroscopic and microscopic levels, HD has the greatest influence on the swelling degree, mechanical strength, and hydrogen nuclear magnetic relaxation. On the other hand, MWs between 31 and 130 KDa do not significantly change the previous properties.

Specifically, the higher the HD of the PVA, the higher the crosslinking density of the gel and, consequently, the resistance to external stresses is greater. However, MW significantly affects the viscosity of the starting PVA solution because the lower the MW of the PVA, the lower the viscosity of the solution. The mechanical strength of the gel reflects this behavior, especially in the case of PVA solutions (*4-100*; *8-100*; *13-100*; *18-100*) and, accordingly, by measuring the viscosity of the solution, it is possible to achieve an idea of gel strength. At the dosimetric level, PVA 20:98, with a higher HD, showed a 5.0% lower optical sensitivity than other PVAs, and, moreover, it promotes the auto-oxidation process of Fe^2+^ ions, suggesting that HD is also a key factor to take into consideration in this case. However, the auto-oxidation process is also affected by low MW.

In conclusion, the composition of FG can be effectively modulated by adjusting the mixture of PVA, allowing for closer mimicry of the diverse consistencies found in human tissues and maintaining the radiological tissue equivalent [24]. When designing dose measures or developing medical imaging techniques, it is crucial to use materials that mimic human tissue’s response to radiation accurately [49]. Therefore, tissue equivalence ensures that the dose delivered to a phantom or simulated tissue closely resembles that in real human tissue, enabling more accurate dosimetry and assessment of the effects of radiation. This concept is vital in medical physics for calibrating radiation beams, determining optimal imaging protocols, and assessing radiation risks in different scenarios [50].

Importantly, this modulation does not compromise the dosimetric response of the dosimeter. However, it is noteworthy that utilizing PVA with either excessively low molecular weight or a high degree of hydrolysis may potentially compromise the temporal stability of the dosimetric response. Therefore, a careful selection of PVA parameters is crucial to strike a balance between achieving tissue-like characteristics and maintaining dosimetric stability over time.

## 4. Materials and Methods

### 4.1. Materials

Poly(vinyl-alcohol) (C_2_H_4_O) KURARAY POVAL™ types 4:88 (MW~31 KDa, HD~88%) 8:88 (MW~67 KDa, HD~88%), 13:88 (MW~94 KDa, HD~88%), 18:88 (MW~130 KDa, HD~88%), and 20:98 (MW~125 KDa, HD~98%) were purchased from Kuraray Europe GmbH [51]. KURARAY POVAL™ is a partially saponified PVA with a low ash content (≤0.5%). Glutaraldehyde (GTA solution 25% *v*/*v* in water) (C_5_H_8_O_2_) was purchased from Sigma Aldrich. Sulfuric acid (SA) (H_2_SO_4_) was purchased from VWR. Ferrous ammonium sulfate hexahydrate (FAS) ((NH_4_)_2_Fe(SO_4_)_2_*6H_2_O) was purchased from Carlo Erba, while Xylenol Orange tetra-sodium sodium salt (XO) (C_31_H_28_N_2_Na_4_O_13_S) was obtained from Riedel-de Haën. All batches of hydrogels were prepared using solvents of analytical reagent grade and ultrapure water (resistivity 18.2 MΩ·cm, Milli-Q Direct, EMD Millipore, Darmstadt Germany).

### 4.2. Hydrogels Preparation

All PVA stock solutions were prepared by dissolving under the magnetic stirring of 10.6 g of PVA in 75 mL of ultrapure water at 70 °C for 40 min for PVA 4:88, 8:88, 13:88, and 18:88 or at 90 °C for 1 h for PVA 20:98. The final concentration of the PVA stock solutions was 12.4% *w*/*w*. After the complete dissolution, the PVA solution was left to cool down at room temperature (25.0 ± 0.5 °C). PVA hydrogels were obtained by adding 12.8 mL of 98 mM SA solution to 36.7 g of PVA mixture solution. PVA 18:88 was selected as a reference due to the fact that it is the one most used in our research group [52,53]. The details of the various PVA mixtures are summarized in Table 6. The sample names (in italics) were identified as follows: the first number (*4-*, *8-*, *13-*, *18-*, and *20-*) refers to the PVA type (4:88, 8:88, 13:88, 18:88, and 20:98, respectively), the second number, separated from the first by a dash, indicates the percentage by weight of PVA indicated by the first number. The remaining percentage of polymer is PVA of type 18:88. The final concentration of PVA was 9.1% *w*/*w*. Lastly, GTA (483 μL) was added under magnetic stirring. The final concentrations of SA and GTA in the gel were 25.0 mM and 26.5 mM, respectively. After 1 min of stirring to achieve homogeneity, the solution was poured into molds to obtain the desired shape. After complete gelation, all of the hydrogels were maintained in a refrigerator at a temperature of 6 °C.

**Table 6:** Details of the PVA mixture in the hydrogel samples.

Sample ID	PVA Mixture				
	% 4:88 *MW*~31 kDa *HD*~88%	% 8:88 *MW*~67 kDa *HD*~88%	% 13:88 *MW*~94 kDa *HD*~88%	% 20:98 *MW*~125 kDa *HD*~98%	% 18:88 *MW*~130 kDa *HD*~88%
*4-100*	100				
*8-100*		100			
*13-100*			100		
*18-100*					100
*20-100*				100	
*4-10*	10				90
*4-20*	20				80
*4-30*	30				70
*4-40*	40				60
*4-50*	50				50
*8-10*		10			90
*8-20*		20			80
*8-30*		30			70
*8-40*		40			60
*8-50*		50			50
*13-10*			10		90
*13-20*			20		80
*13-30*			30		70
*13-40*			40		60
*13-50*			50		50
*20-10*				10	90
*20-20*				20	80
*20-30*				30	70
*20-40*				40	60
*20-50*				50	50

### 4.3. Determination of the Viscosity

The apparent viscosity (*η*) of the precursor PVA solutions (Table 6) was measured using a viscometer (ROTAVISC me-vi Complete, IKA^®^-Werke GmbH & Co. KG, Staufen, Germany) equipped with the reducer VOLS-1. The measurements were conducted by varying the temperature (from 15 °C to 30 °C) and the rotation speed (from 20 to 200 rpm). The viscometer was connected to a recirculating bath in order to observe a change in the viscosity as a function of temperature. These measurements were carried out using a customized water bath, as described in [54], and implemented in order to maintain the temperature of the samples at controlled values. The heating rate was about 1 °C/min., the viscosity was recorded at regular temperature steps, and the rotational speed was set at 150 rpm. The viscosity and the shear stress of the PVA mixtures were measured at various shear rates at 27.0 °C. The temperature was maintained within ±0.1 °C.

### 4.4. Uniaxial Compression Mechanical Tests

Uniaxial compression tests were carried out on cylindrical specimens (h = 8 mm; Ø = 16 mm) at room temperature (25.0 ± 0.5 °C). Tests were performed using a Universal Test Machine (Lloyd Instruments) equipped with a 50 N cell load at a displacement rate of 10 mm/min. A safety stop was imposed at 40 N to avoid damaging the cell load. Strain (ε) was calculated as the ratio of the crosshead displacement to the initial sample height. Stress (σ) was calculated as the ratio of the measured force to the un-deformed sample cross-section. Young’s modulus (*E*) was obtained from the initial portion of the stress−strain curve (strain up to 10%).

### 4.5. FTIR Measurements

The FTIR spectra were recorded using the FTIR FAME Analyzer, which was composed of a Frontier ATR FTIR spectrometer, on samples in a xerogel form, operating in free air conditions and at room temperature (25.0 ± 0.5 °C). The spectra were acquired from 4000 to 500 cm^−1^ at a resolution of 4 cm^−1^ with 120 scans per sample. FTIR measurements were performed on *4-100*, *8-100*, *13-100*, *18-100*, *20-100*, *4-50*, *8-50*, *13-50*, and *20-50* samples.

### 4.6. Gel Fraction Determination

PVA hydrogel samples were molded into a cylindrical shape (h = 10 mm; Ø = 22 mm) for gel fraction (*GF*%), swelling (*SW*%), and water loss (*WL*%) measurements.

The samples were placed in an oven (Bio-Optica oven SVN1790) at 37.0 ± 0.5 °C until a constant weight was reached before *GF*% measurements. Then, each sample was immersed in ultrapure water at room temperature for 4 days. Subsequently, the immersed sample was removed from distilled water and dried at 37 ± 0.5 °C until a constant weight was reached. Therefore, the *GF*% could be calculated as follows:

(2) $$GF\%=\frac{W_{f}}{W_{i}}\times 100$$

where *W_i_* and *W_f_* are the weights of the xerogels before and after the immersion, respectively.

### 4.7. Swelling Degree Measurements

*SW*% determinations were carried out in ultrapure water. All of the samples were dried before immersion at 37.0 ± 0.5 °C for 48 h. The equilibrium swelling degree (*SW*%) was determined as follows:

(3) $$SW\%=\frac{W_{eq}-W_{i}}{W_{i}}\times 100$$

where *W_i_* is the weight of the samples before immersion, and *W_eq_* is the weight of the sample at equilibrium water content.

### 4.8. Water Loss Determination

To perform *WL*% measurements, each sample was placed at room temperature (25.0 ± 0.5 °C) and weighed at set times. The water loss was determined as follows:

(4) $$WL\%=(1-\frac{W_{t}}{W_{i}})\times 100$$

where *W_t_* and *W_i_* are the weights of the samples at the setting and initial time, respectively.

### 4.9. Contact Angle Analyses

Contact angle analyses were performed using the FTA1000 Analyzer System. The images and the contact angle calculation were carried out using FTA32 video software. The hydrogel samples were left to cure at 37 °C for one night, and then the measurements were conducted at room temperature on at least five different locations of the sample. The size of the deposited drop was 150 μL. Contact angle measurements were performed on *4-100*, *8-100*, *13-100*, *18-100*, *20-100*, *4-50*, *8-50*, *13-50*, and *20-50* samples.

### 4.10. ^1^H NMR Relaxometry Measurements

^1^H NMR measurements were performed using a standard homemade broadband spectrometer working at 31.6 MHz, corresponding to a magnetic field of 0.74 Tesla. The coil was designed to adapt perfectly to the sample holder (TD-NMR glass tubes 10 mm) in order to maximize the filling factor and, consequently, increase the signal-to-noise ratio and thus perform fast measurements. Both longitudinal (T_1_) and transverse (T_2_) relaxation times were acquired using standard pulse sequences, namely a saturation recovery for T_1_ measurements and Carr–Purcell–Meiboom–Gill for T_2_ measurements (pulse: π/2 = 1.9 μs, repetition time: RT ≥ 1.5 s). The echo signal integrated over time has been plotted as a function of different delay times, as usual in the T_2_–decay curve.

For this study, we opted to examine pure samples and 50/50 mixtures. The choice was made considering that the measurements necessitate extended periods and could be susceptible to the release of water from the matrix. ^1^H NMR measurements were performed on *4-100*, *8-100*, *13-100*, *18-100*, *20-100*, *4-50*, *8-50*, *13-50*, and *20-50* samples. The T_1_ experimental curves were fitted with a model that considers a single exponential recovery of the magnetization, and the T_2_ experimental curves were fitted with a model that implements the sum of different exponential decays (up to 3 components) by means of Microcal Origin software. In the case of T_2_ curves, the Uniform PENalty (UPEN) inversion algorithm, which performs the inverse Laplace transform on multi-exponential relaxation curves, giving back the T_2_ times distribution, was also used.

### 4.11. Dosimetric Application

#### 4.11.1. Fricke Hydrogel Dosimeter Preparation

The PVA-GTA-FG dosimeters were prepared according to the hydrogel preparation described in Section 4.2 by infusing a “Fricke solution” composed of SA, FAS, and XO instead of the SA solution. The gel solution was poured into standard spectrophotometry cuvettes (10 mm optical path), closed with plastic stoppers, and sealed with Parafilm™. After the preparation, PVA-GTA-FG dosimeters were kept refrigerated at a temperature of 6 °C for one day and brought back to room temperature 1 h before irradiation.

The final PVA-GTA-FG was composed of PVA (9.1% *w*/*w*), SA (25.0 mM), FAS (0.50 mM), XO (0.165 mM), and GTA (26.5 mM).

#### 4.11.2. Fricke Hydrogel Dosimeter Irradiation

Irradiation was carried out with a linear accelerator (LINAC) Varian Clinac-Trilogy (Varian Medical Systems, CA, USA) at “Fondazione IRCCS Istituto Nazionale dei Tumori” of Milano (Italy). The dose–response of the PVA-GTA-FG dosimeters was studied by irradiating the samples at increasing doses up to 18 Gy with 6 MV X-ray beams using dose rates of 9.90 cGy/s.

The LINAC was calibrated following the IAEA TRS-398 code of practice (IAEA 2000, Vienna, Austria). In all of the experiments, at least three samples of the same batch were irradiated simultaneously. The samples were placed horizontally within a solid water slab phantom, with the central plane of the cuvettes at a depth of 5 cm from the phantom surface. All of the irradiation procedures were performed using a Source Detector Distance (SDD) of 100 cm (i.e., the distance between the source and the central plane of the cuvettes). The uniformity of dose within the cuvette volumes was achieved by using a 20 cm × 20 cm field size at the position of the dosimeters. The details of the irradiation geometry are available elsewhere [53].

#### 4.11.3. Optical Absorbance Measurements

For dosimetric characterization, the optical absorbance (OA) measurements were performed using a Cary 100 UV-Vis spectrophotometer (Agilent Technologies, Santa Clara, CA, USA) in the wavelength range of 360–720 nm with steps of 1 nm. Since in gel dosimetry, the absorbed dose is correlated to the optical absorbance variation Δ(OA) of the dosimeters, the OA spectra of the samples were collected before and after the irradiation [55]. For quantitative analysis, the integral of optical absorbance variation Σ(OA), i.e., the sum of Δ(OA) between 480 and 620 nm, was chosen as the dosimetric parameter. Furthermore, in order to investigate self-oxidation phenomena, three unirradiated PVA-GTA-FG dosimeters of each different preparation (see Table 6) were subjected to thermal stress proceedings using a thermalized water phantom (described in [54]). Starting from refrigerator temperature, the samples were heated up to 27.0 ± 0.5 °C. After a thermalization time of 15 min, the OA spectra of these samples were measured at regular times t_i_, starting from t_0_ = 0 up to t_f_ = 120 min, at approximately 15 min steps.

## Figures and Tables

**Table 1:** Young’s modulus of PVA hydrogels.

Young’s Modulus	Sample ID				
	*4-100*	*8-100*	*13-100*	*18-100*	*20-100*
Mean ± 1SD [kPa]	14.1 ± 3.2	23.2 ± 3.3	39.6 ± 4.0	38.3 ± 5.7	72.6 ± 4.2

**Table 2:** Contact angles and correlated error for each sample.

Sample ID	Contact Angle ± SD (°)
*18-100*	57.78 ± 6.36
*4-100*	44.61 ± 5.78
*4-50*	37.02 ± 4.58
*8-100*	59.48 ± 11.77
*8-50*	55.61 ± 11.90
*13-100*	54.07 ± 1.65
*13-50*	68.55 ± 6.68
*20-100*	49.65 ± 5.86
*20-50*	64.78 ± 8.91

**Table 3:** T_1_ values and correlated error for each sample obtained by means of a fitting model that considers a single exponential recovery of magnetization.

Sample ID	T_1_ ± SD (s)
*18-100*	1.535 ± 0.123
*4-100*	1.563 ± 0.125
*4-50*	1.588 ± 0.127
*8-100*	1.550 ± 0.124
*8-50*	1.567 ± 0.125
*13-100*	1.460 ± 0.117
*13-50*	1.411 ± 0.113
*20-100*	1.411 ± 0.113
*20-50*	1.468 ± 0.117
Mean	1.506 ± 0.023

**Table 4:** Nuclear relaxation times T_2_ and related errors obtained using Equation (1) in the fitting procedure.

Sample ID	T_2 fast_ ± SD (s)	Weight of T_2 fast_ (%)	T_2 medium_ ± SD (s)	Weight of T_2 medium_ (%)	T_2 slow_ ± SD (s)	Weight of T_2 slow_ (%)
*18-100*	0.036 ± 0.003	5.3	0.626 ± 0.050	14.6	1.667 ± 0.134	80.1
*4-100*	0.021 ± 0.002	4.7	-	-	1.601 ± 0.128	95.3
*4-50*	0.009 ± 0.001	2.9	-	-	1.686 ± 0.135	97.1
*8-100*	0.025 ± 0.002	5.0	0.638 ± 0.051	18.7	1.726 ± 0.138	76.3
*8-50*	0.018 ± 0.002	6.0	0.326 ± 0.026	3.7	1.631 ± 0.130	90.3
*13-100*	0.018 ± 0.002	4.9	0.445 ± 0.036	0.5	1.545 ± 0.124	94.6
*13-50*	0.009 ± 0.001	3.7	-	-	1.565 ± 0.125	96.3
*20-100*	0.019 ± 0.002	4.8	-	-	1.140 ± 0.091	95.2
*20-50*	0.021 ± 0.002	4.6	-	-	1.347 ± 0.108	95.4

**Table 5:** Nuclear relaxation times T_2_ and the corresponding percentage weights obtained using the Uniform PENalty (UPEN) inversion algorithm that performs the inverse Laplace transform on multi-exponential relaxation curves.

Sample ID	T_2 fast_ (s)	Weight of T_2 fast_ (%)	T_2 medium_ (s)	Weight of T_2 medium_ (%)	T_2 slow_ (s)	Weight of T_2 slow_ (%)
*18-100*	0.019	4.7	0.289	6.4	1.550	88.9
*4-100*	0.017	5.1	-	-	1.600	94.9
*4-50*			Not Resolved			
*8-100*	0.021	5.1	0.525	12.7	1.630	82.2
*8-50*	0.013	7.5	0.332	3.4	1.630	89.1
*13-100*	0.019	4.4	-	-	1.540	95.6
*13-50*	0.009	3.3	-	-	1.560	96.7
*20-100*	0.017	4.8	-	-	1.140	95.2
*20-50*	0.014	4.9	-	-	1.320	95.1

## Data Availability

All data and materials are available on request from the corresponding author.

## Supplementary Materials

The following supporting information can be downloaded at: https://www.mdpi.com/article/10.3390/gels10030172/s1, Figure S1: Viscosity profile of PVA mixture solutions at increasing temperature and at a share rate of 150 rpm; Figure S2: FTIR spectra of a) PVA powders and b) of xerogels: 4-100, 4-50, 18-100, 8-100, 8-50, 13-100, 13-50, 20-100 and 20-50; Figure S3: Evaporation profiles of PVA hydrogel samples as function of time; Figure S4: Contact angle images of PVA hydrogels: 4-100, 4-50, 18-100, 8-100, 8-50, 13-100, 13-50, 20-100 and 20-50; Figure S5: Longitudinal nuclear relaxation decay curves of the samples: 4-100, 4-50, 18-100, 8-100, 8-50, 13-100, 13-50, 20-100 and 20-50; Table S1: Dosimetric parameters.
