# Supplementary material for: Microscopic and Macroscopic Characterization of Hydrogels Based on Poly(vinyl-alcohol)–Glutaraldehyde Mixtures for Fricke Gel Dosimetry
Source: Gels. 2024 Feb 28;10(3):172. doi: 10.3390/gels10030172 (PMC10970131; doi:10.3390/gels10030172)
Supplement: Supplementary file 1 [file gels-10-00172-s001.zip › gels-2853566-supplementary.pdf]

gels

MDPI

Microscopic and macroscopic characterization of hydrogels based on Poly(vinyl-alcohol)–Glutaraldehyde mixtures for Fricke gel dosimetry

Silvia Locarno*, Paolo Arosio, Francesca Curtoni, Marco Piazzoni, Emanuele Pignoli, Salvatore Gallo

Supporting Information

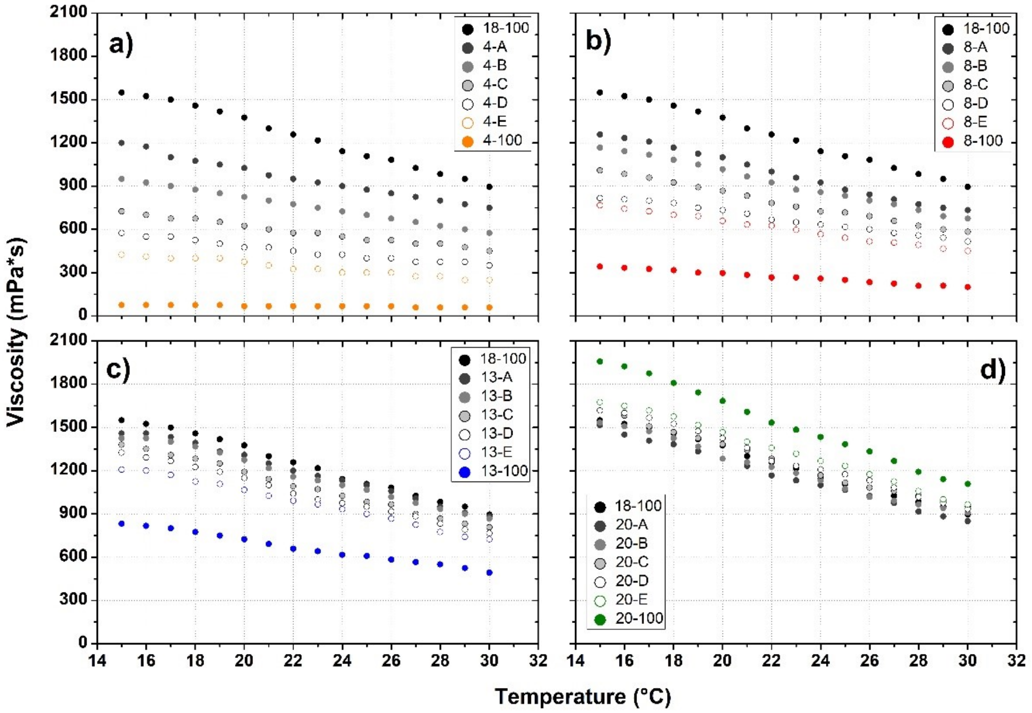

Figure S1 - Viscosity profile of PVA mixture solutions at increasing temperature and at a share rate of 150 rpm.

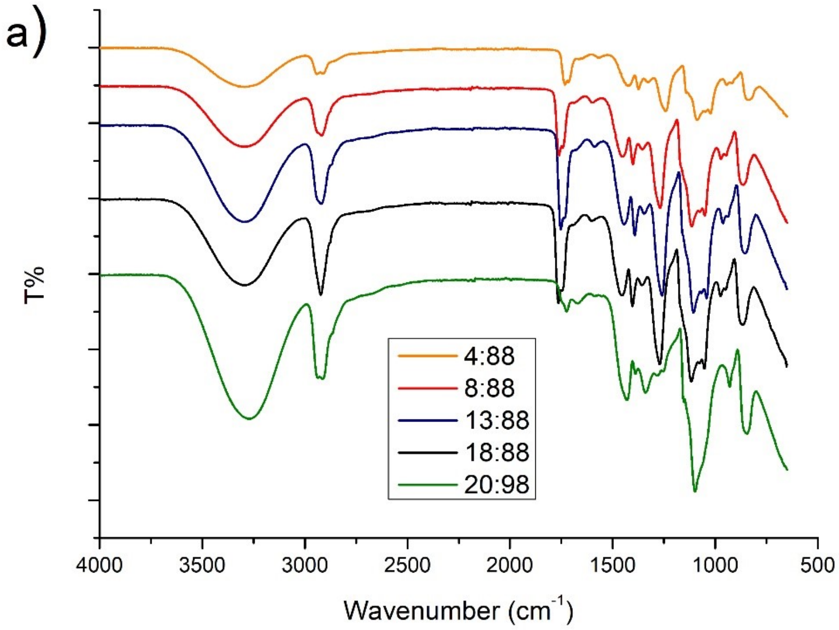
a)
T%
4:88
8:88
13:88
18:88
20:98
4000
3500
3000
2500
2000
1500
1000
500
Wavenumber (cm-1)

8

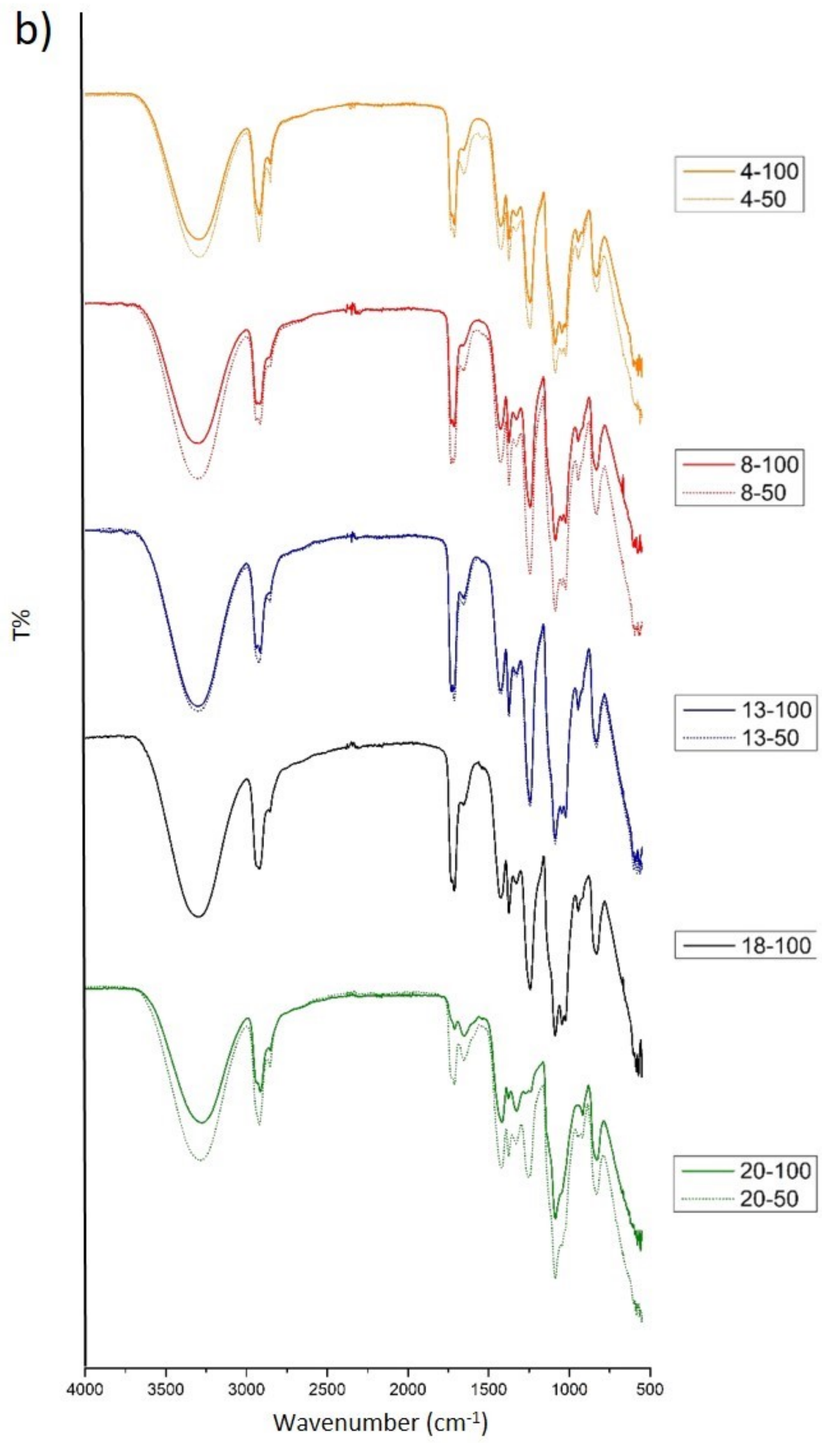

Figure S2 – FTIR spectra of a) PVA powders and b) of xerogels: *4-100*, *4-50*, *18-100*, *8-100*, *8-50*, *13-100*, *13-50*, *20-100* and *20-50*.

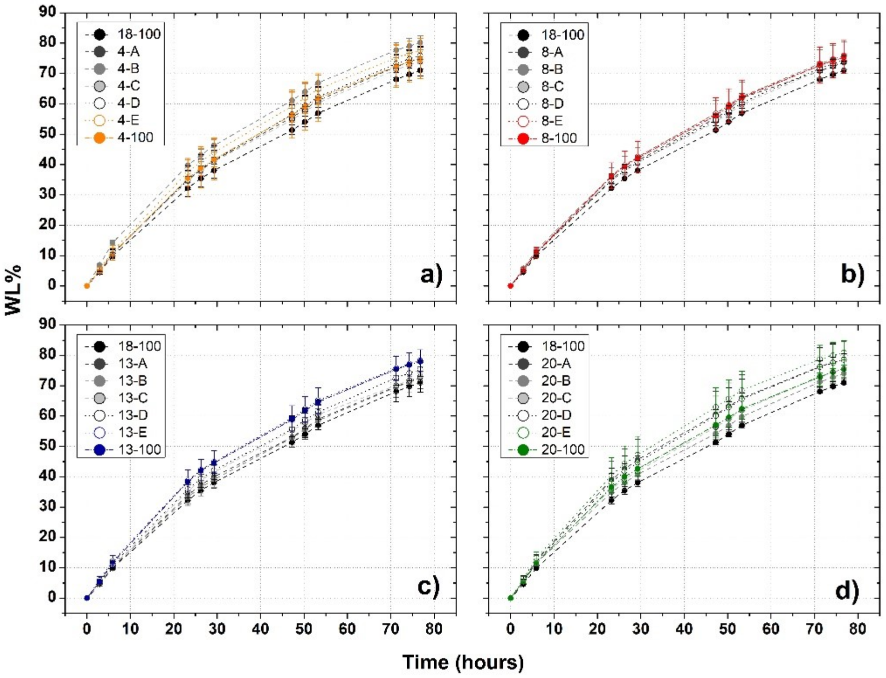

Figure S3 - Evaporation profiles of PVA hydrogel samples as function of time.

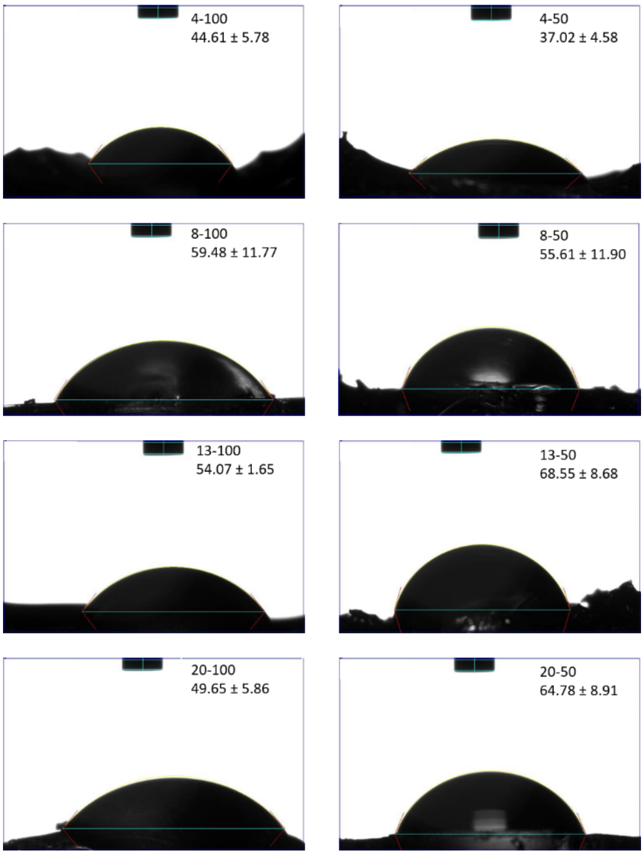

Figure S4 – Contact angle images of PVA hydrogels: *4-100*, *4-50*, *18-100*, *8-100*, *8-50*, *13-100*, *13-50*, *20-100* and *20-50*

Table S1 - Dosimetric parameters.

Sample	Dose Sensitivity	
4-100	7.86	±0.08
8-100	7.75	±0.14
13-100	7.60	±0.08
18-100	7.65	±0.07
20-100	7.32	±0.09
4-10	7.57	±0.09
4-20	7.60	±0.07
4-30	7.70	±0.07
4-40	7.61	±0.07
4-50	7.64	±0.08
8-10	7.59	±0.02
8-20	7.64	±0.03
8-30	7.53	±0.07
8-40	7.56	±0.07
8-50	7.59	±0.06
13-10	7.57	±0.09
13-20	7.60	±0.07
13-30	7.70	±0.07
13-40	7.61	±0.07
13-50	7.64	±0.08
20-10	7.66	±0.11
20-20	7.43	±0.09
20-30	7.37	±0.10
20-40	7.33	±0.08
20-50	7.32	±0.09

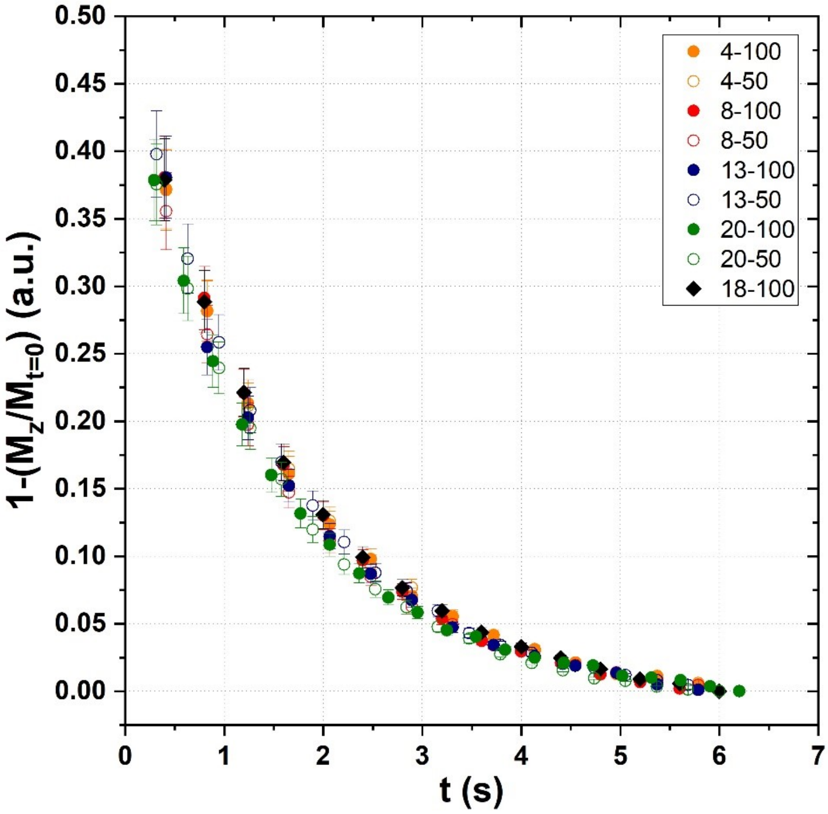

Figure S5 - Longitudinal nuclear relaxation decay curves of the samples: *4-100*, *4-50*, *18-100*, *8-100*, *8-50*, *13-100*, *13-50*, *20-100* and *20-50*.
